# The dynamic changes of main cell types in the microenvironment of sciatic nerves following sciatic nerve injury and the influence of let-7 on their distribution

**DOI:** 10.1039/c8ra08298g

**Published:** 2018-12-10

**Authors:** Tianmei Qian, Pan Wang, Qianqian Chen, Sheng Yi, Qianyan Liu, Hongkui Wang, Shiyuan Wang, Wenqing Geng, Zihao Liu, Shiying Li

**Affiliations:** Key Laboratory of Neuroregeneration of Jiangsu, Ministry of Education, Co-innovation Center of Neuroregeneration, Nantong University Nantong Jiangsu 226001 China lisy0379@ntu.edu.cn +86-513-85511585 +86-513-85511585

## Abstract

Schwann cells (SCs), fibroblasts and macrophages are the main cells in the peripheral nerve stumps. These three types of cell play important roles in regulating the regeneration microenvironment and improving the regeneration effect through a variety of manual interventions. However, the dynamic distribution of these cells during the different stages of peripheral nerve regeneration remain unclear. In this study, we systematically explored the number/ratio and distribution changes of SCs, fibroblasts, and macrophages at different time points after sciatic nerve injury. Moreover, considering that let-7 is an ideal molecule for regulating the regeneration environment, we further studied the entrance and influence of let-7 antagomir on these three main cell types. Collectively, our current study revealed the cell basis of the microenvironment of peripheral nerve regeneration and indicated that let-7 could modify the regenerative microenvironment by regulating the number/ratio and distribution of SCs, fibroblasts, and macrophages. Our study would help to open a new potential therapeutic window for peripheral nerve injury.

## Abstract 2

Schwann cells (SCs), fibroblasts and macrophages are the main cells in the peripheral nerve stumps.

## Graphical abstract

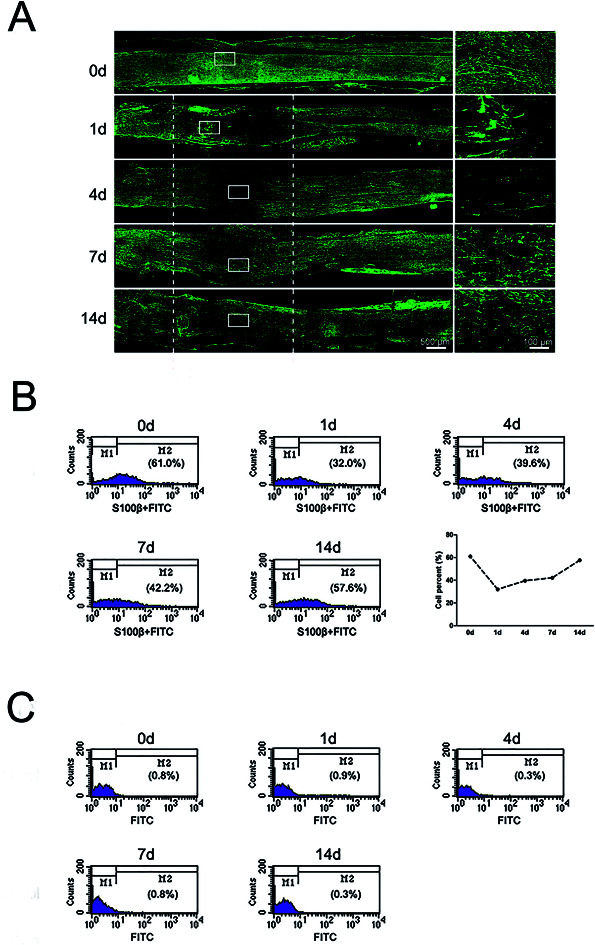

## Introduction

Peripheral nerve injury is usually caused by accidents, natural disaster, war damage, other trauma, as well as iatrogenic side effects of surgery.^1^ Nowadays, peripheral nerve injury has become a common clinical problem around the world. It may result in total or partial loss of motor, sensory, and autonomic functions, which significantly impairs the patients' quality of life and causes an enormous financial burden.^2,3^ Fortunately, unlike the central nervous system (CNS) that fails to regenerate spontaneously after injury, the peripheral nervous system (PNS) has an intrinsic regenerative ability in response to injury. However, the recovery effects of PNS are often unsatisfactory.^4^ Peripheral nerve regeneration involves precise coordination and complex interactions among a number of cellular and molecular events to establish an optimal microenvironment for axonal regrowth and target reinnervation.^5,6^

The microenvironment of peripheral nerve regeneration is a complex process that involves multi-cellular response, such as Schwann cells (SCs), fibroblasts, and macrophages, which are the main cell types involved in regulating nerve regeneration.^7–9^ As is well known, peripheral nerve injury leads to Wallerian degeneration in the distal stump, which serves to create a favorable microenvironment for axonal regeneration.^10,11^ The intrinsic degeneration of injured axons triggers inflammatory response that helps to generate an environment to support regeneration by removing inhibitory myelin and up-regulating molecules like cytokines, chemokines, and transcription factors.^8,12^ The inflammatory response involves multiple types of cells, including SCs, fibroblasts, macrophages, endothelial cells, and effector molecules produced by cells with both positive and negative effects.^13,14^ SCs are the major glial cells of the PNS and participate in many biological functions of the PNS.^10^ Upon peripheral nerve injury, SCs dedifferentiate, proliferate, migrate, and form bands of Bungner to guide axonal regrowth.^15,16^ SCs also help removing myelin debris and secreting multiple neurotrophic factors, cytokine, adhesion molecules and extracellular matrix molecules to benefit the construction of a benefit microenvironment for axonal regrowth.^17^ Following peripheral nerve injury, large numbers of fibroblasts accumulate at the injury site and secrete new extracellular matrix components to promote axonal regeneration. Fibroblasts also initiate tissue reconstruction by orchestrating SC sorting and promoting SC migration.^18^ Macrophages, the most notable immune cells, accumulate at the degenerating nerve stumps to remove myelin and axon debris.^12^ They not only contribute to debris clearance, but also are educated by the local injured microenvironment to release a large number of axonal regeneration-related factors, polarized to an anti-inflammatory phenotype and stimulated regeneration near the axotomized neuronal cell bodies, thus contributing to axonal regeneration.^19^ Following nerve repair, to reach the final goal of nerve regeneration, axons must have the intrinsic ability to regrow, the regeneration environment must support regrowth, and the target tissues must achieve new axons.^20^ Thus, an appropriate microenvironment is beneficial for the regeneration of peripheral nerves, and understanding the underlying cellular mechanisms is of great importance. However, until now, there is no systematic study about the dynamic changes of SCs, fibroblasts, and macrophages at different stages during peripheral nerve regeneration.

MicroRNAs (miRNAs) are a class of endogenous, small non-coding RNA molecules that serve as post-transcriptional regulators of gene expression.^21^ They have been demonstrated to play pivotal roles in many physiological and pathological processes, such as cell differentiation, proliferation, migration, apoptosis, and morphogenesis.^22^ Additionally, emerging evidences indicate that miRNAs significantly affect the biological behaviors of neurons and SCs during peripheral nerve regeneration, such as survival maintenance, axonal regrowth, and phenotype modulation of SCs.^23–25^ Previous study identified that inhibiting let-7 could promote SC migration and axon outgrowth within a regenerative microenvironment after peripheral nerve injury.^3^ In addition, let-7 could regulate inflammatory response by targeting inflammatory factors including interleukin-6 (IL-6) and interleukin-10 (IL-10),^26,27^ which also involved in regulating cell apoptosis, cell differentiation, cell proliferation, cell migration, and myelin formation.^28–31^ In a word, let-7 widely regulates important biological events related to the microenvironment of nerve regeneration.

Nerve growth factor (NGF), a family of neurotrophic factors called neurotrophins, contributes to the survival and maturation of developing neurons and SCs and plays important roles in axonal regrowth and myelination after injury.^32^ Therefore, NGF has been widely used in tissue engineered nerves.^33–36^ However, the clinical applications of NGF are still limited by several constraints, such as unable to pass blood-nerve barrier, short circulating half-life, deleterious side effects, and so on.^37^ The application of miRNA in tissue engineering has also been widely recognized,^38–40^ our previous study showed that let-7 could affect SC phenotype by directly suppressing the protein translation of NGF.^3^ Therefore, let-7 acts as an ideal molecule for constructing molecule-integrated tissue engineering nerve, which suggested a potential therapy for repair of peripheral nerve injury. The dynamic changes of SCs, fibroblasts, and macrophages are the endogenous environmental basis for the construction of molecular tissue engineering nerves. But it is not clear the entrance and influence of let-7 on these three cell types during peripheral nerve injury and regeneration.

In this study, we aimed to investigate the dynamic distributions and proportion changes of SCs, fibroblasts, and macrophages following sciatic nerve injury. We also investigated the entrance of let-7 antagomir into these three main cell types and the influence of let-7 antagomir on their proportions and distributions. This would provide a novel potential therapeutic method for peripheral nerve injury by the application of let-7 on tissue engineering nerve.

## Materials and methods

### Construction of animal surgery and tissue preparation

Male Sprague-Dawley (SD) rats (Experimental Animal Center of Nantong University), weighing between 180 and 220 g, were used in the experiments. Rats were divided into 5 groups of 15 rats each randomly and were anaesthetized by an intraperitoneal injection of complex narcotics (85 mg kg^−1^ trichloroacetaldehyde monohydrate, 42 mg kg^−1^ magnesium sulfate, and 17 mg kg^−1^ sodium pentobarbital), and the sciatic nerve was exposed through an incision on the mid-thigh of left hind limb. The sciatic nerve was crushed twice (15 seconds each time, 3 seconds interval) with a hemostatic forceps. After the surgical incisions were closed, animals were housed in temperature and humidity controlled environment, maintained under a 12 : 12 hour light dark cycle, and were allowed free access to water and food. A 3 mm long crushed nerve, together with both nerve ends, was harvested at 0, 1, 4, 7, or 14 days after nerve crush for immunohistochemistry. A crushed nerve was harvested at different time points for flow cytometry analysis.

For *in vivo* experiments, the crush site was injected with let-7d antagomir or control, respectively. At 1 and 4 days after surgery, rats were killed and their sciatic nerves were harvested for immunohistochemistry, immunofluorescence, and flow cytometry analysis.

All experimental protocols involving animals were conducted in accordance with Institutional Animal Care guidelines of Nantong University, China (animal license number: SCXK (Su) 2014-0001 and SYXK (Su) 2012-0031) and approved ethically by the Administration Committee of Experimental Animals, Jiangsu Province, China (approval no. 20150304-004).

### Immunohistochemistry and immunofluorescence

The harvested sciatic nerve segments were fixed in 4% paraformaldehyde and dehydrated in sucrose solution. Cryostat sections of 12 μm were cut and placed on slides that were frozen at −80 °C. For staining, slides were thawed and rinsed in PBS, followed by permeabilization for 1 hour in 5% goat serum, 1% BSA, and 0.3% Triton X-100. Then, slides were incubated with primary specific antibodies: rabbit anti-S100β antibody (1 : 100, Abcam, Cambridge, MA), rabbit anti-P4HB antibody (1 : 100, Abcam), mouse anti-CD68 antibody (1 : 200, Abcam), and mouse anti-NF200 antibody (1 : 100, Sigma, St. Louis, MO) at 4 °C for 12 hours, respectively. After rinsing, slides were incubated with corresponding secondary antibodies: Alexa Fluor 488 donkey anti-rabbit IgG (1 : 500, Proteintech Group, Chicago, IL) and Alexa Fluor 488 donkey anti-mouse IgG (1 : 500, Proteintech Group) at room temperature for 2 hours. Finally, the samples were observed and photographed under a fluorescence microscope (Zeiss, Germany).

The sciatic nerve segments were harvested at 4 days after surgery, trypsinized, and then plated at a density of 5 × 10^5^ cells per ml on poly-l-lysine-coated 24-well plates. The cells were cultured in Dulbecco's modified Eagle's medium (DMEM) supplemented with 10% fetal bovine serum (FBS) at 37 °C in a humidified 5% CO_2_ incubator. After 24 hours, the cultured cells were fixed in 4% paraformaldehyde at room temperature for 15 minutes, and then immunostained with primary antibodies: rabbit anti-S100β antibody, rabbit anti-P4HB antibody, and mouse anti-CD68 antibody, respectively, followed by reaction with fluorescently labeled secondary antibodies: Alexa Fluor 488 donkey anti-rabbit IgG and Alexa Fluor 488 donkey anti-mouse IgG. Images were taken under a fluorescence microscope.

### Flow cytometry analysis

Flow cytometry analysis was operated for measuring the proportions of SCs, fibroblasts, macrophages and the influence of entrance and proportions of let-7 antagomir on these three cell types in sciatic nerve by using corresponding antibodies. The experiments were performed according to the manufacturer's instructions. Briefly, sciatic nerve segments were harvested at the indicated time points post-injury, trypsinized, and then fixed in medium A at room temperature for 15 minutes. Fixed cells were centrifuged, washed, and incubated with primary antibodies: rabbit anti-S100β antibody (1 : 100, Proteintech Group), rabbit anti-P4HB antibody (1 : 100, Proteintech Group), and rabbit anti-CD68 antibody (1 : 100, Proteintech Group) at room temperature for 1 hour, respectively. Then, cells were stained with Alexa Fluor 488 donkey anti-rabbit IgG for 1 hour in dark at room temperature. Finally, cells were washed, resuspended in PBS containing 5% FBS and analyzed by flow cytometer (BD Bioscience, San Jose, CA).

## Results

### Distributions and proportion changes of SCs after sciatic nerve injury

**Fig. 1:**
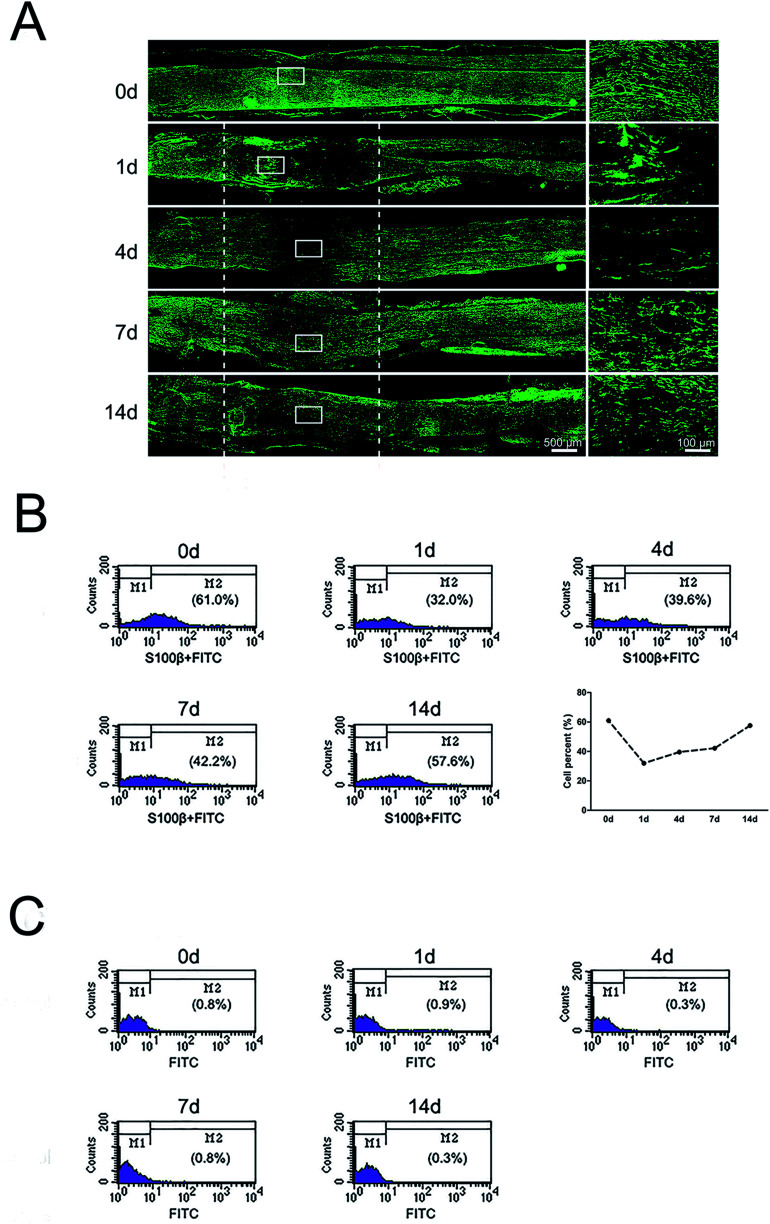
The dynamic distributions and proportion of SCs following sciatic nerve crush. (A) Immunostaining with anti-S100β (green) of longitudinal sections of sciatic nerve harvested at 0, 1, 4, 7, and 14 days post-nerve crush, respectively. White dotted line in the left image marked the crushed area (3 mm long). Scale bar: 500 μm. High magnifications of white boxed areas in the left image showed the migrating SCs on the frontier. Scale bar: 100 μm. (B) Flow cytometry showing the proportion of SCs at 0, 1, 4, 7, and 14 days post-nerve crush, respectively. Line chart showed SCs percent from flow cytometry. (C) Flow cytometry showing the proportion of negative control at 0, 1, 4, 7, and 14 days post-nerve crush, respectively.

Immunohistochemistry with SC marker anti-S100β was applied to analyze the distributions of SCs in the injured nerve stumps at 0, 1, 4, 7, and 14 days post-sciatic nerve crush. Results showed that SCs formed a multi-layered, membranous myelin sheath around axons in the normal sciatic nerve (0 day) (Fig. 1A). The signals of S100β in the crushed nerve segment were significantly reduced at 1 day post-nerve injury. Meanwhile, signals of S100β were mainly accumulated at the proximal stump at this time point. The signals of S100β were significantly increased and started to migrate into middle part from both injury ends at 4 days post-nerve injury. S100β-positive SCs at crushed nerve segment kept increased and then were distributed at the whole crushed nerve segment at 7 and 14 days post-nerve injury.

To further understand the proportion changes of SCs at 0, 1, 4, 7, and 14 days post-sciatic nerve crush, flow cytometry was used to detect the amount of SCs after sciatic nerve injury. Results showed that SCs constituted 61.0% of nucleated cells in uninjured peripheral nerves. But at 1 day post-sciatic nerve crush, the proportion of SCs dropped to half as compared to that at 0 day post-sciatic nerve crush (control). Subsequently, the proportion of SCs increased at 4, 7 and 14 days post-injury and reached to 57.6% at 14 days post-injury, which was close to the level of uninjured (Fig. 1B). In contrast, the percentages of negative control at each time pint were remain low, indicating the accuracy of flow cytometry results (Fig. 1C).

### Distributions and proportion changes of fibroblasts after sciatic nerve injury

**Fig. 2:**
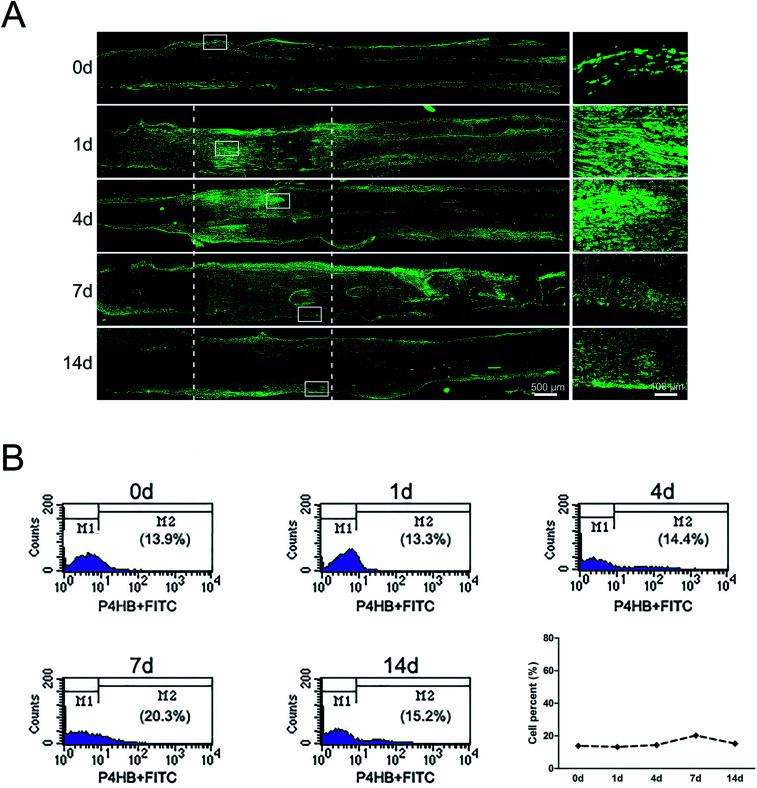
The dynamic distributions and proportion of fibroblasts following sciatic nerve crush. (A) Immunostaining with anti-P4HB (green) of longitudinal sections of sciatic nerve harvested at 0, 1, 4, 7, and 14 days post-nerve crush, respectively. White dotted line in the left image marked the crushed area (3 mm long). Scale bar: 500 μm. High magnifications of white boxed areas in the left image showed the accumulated signals of P4HB. Scale bar: 100 μm. (B) Flow cytometry showing the proportion of fibroblasts at 0, 1, 4, 7, and 14 days post nerve crush, respectively. Line chart showed fibroblasts percent from flow cytometry.

The sciatic nerve was also subjected to immunostaining with fibroblasts marker anti-P4HB. Results showed that in the normal sciatic nerve, fibroblasts mainly localized in the epineurium and perineurium (Fig. 2A). But at 1 day post-sciatic nerve crush, fibroblasts were observed to be accumulated at both nerve injury ends, especially at the proximal nerve stump. Then, fibroblasts migrated from the proximal to the distal part at the crushed nerve segment at 4 days post-sciatic nerve crush. Yet, although the signals of P4HB were distributed in the whole crushed nerve segment, fibroblasts began to migrate to membrane at 7 days post-sciatic nerve crush. Upon to 14 days post-sciatic nerve crush, P4HB-positive fibroblasts gathered in the epineurium and perineurium again as the normal sciatic nerve. In additional, the signals of P4HB were increased from 1 to 7 days after sciatic nerve crush and then decreased at 14 days post-sciatic nerve crush. Consistently, the flow cytometry results confirmed that the proportion of fibroblasts was 13.9% of nucleated cells with uninjured peripheral nerves, reached to 20.3% at 7 days post-injury, but then dropped to 15.2% after 14 days (Fig. 2B).

### Distributions and proportion changes of macrophages after sciatic nerve injury

**Fig. 3:**
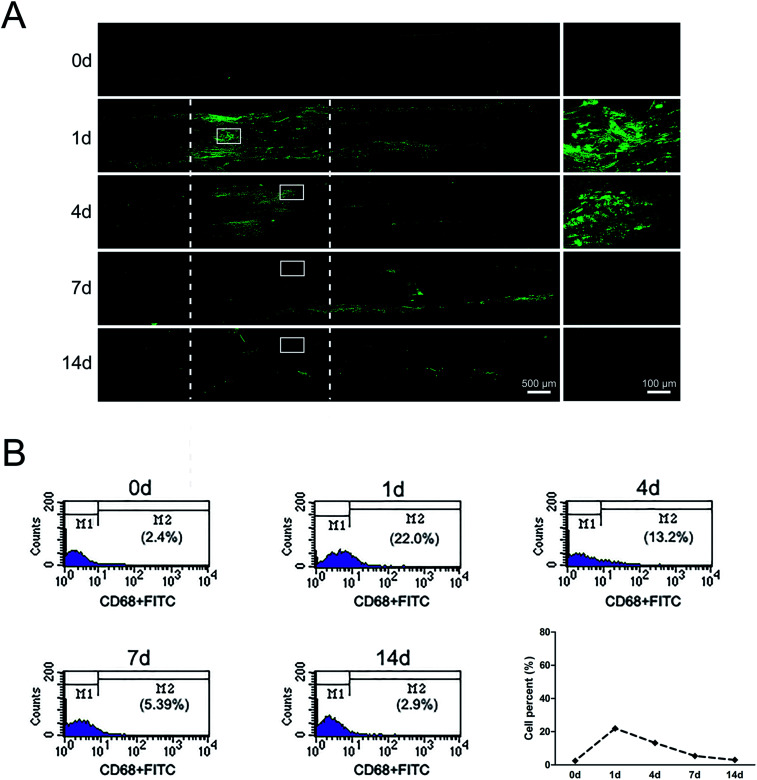
The dynamic distributions and proportion of macrophages following sciatic nerve crush. (A) Immunostaining with anti-CD68 (green) of longitudinal sections of sciatic nerve harvested at 0, 1, 4, 7, and 14 days post nerve crush, respectively. White dotted line in the left image marked the crushed area (3 mm long). Scale bar: 500 μm. High magnifications of white boxed areas in the left image showed the signals of CD68. Scale bar: 100 μm. (B) Flow cytometry showing the proportion of macrophages at 0, 1, 4, 7, and 14 days post nerve crush, respectively. Line chart showed macrophages percent from flow cytometry.

At 1 day post-surgery, immunohistochemical staining of sciatic nerve tissue sections revealed a large number of CD68-positive (macrophages marker) cells in rats underwent sciatic nerve crush injury (Fig. 3A). CD68-positive cells were not nearly detectable in normal sciatic nerve. But the signals of CD68 were significantly increased after nerve injury, especially at 1 day post-injury. At 4 days post-surgery, macrophages were mainly recruited at the distal stump of crushed nerve segment. These results indicated that a large number of macrophages arrived at the sciatic nerve injury site in an early stage within 4 days post-injury. Notably, there existed a morphological change of macrophages at different time point post-nerve injury. At 1 day post-nerve injury, most CD68-positive cells showed a compact and elongated shape while at 4 days post-nerve injury, most CD68-positive cells were large and amoeboid.

Further study about the proportion changes of macrophages at different time points days after sciatic nerve injury were performed by using flow cytometry analysis. Flow cytometry results indicated that the proportion of macrophages only 2.4% in uninjured peripheral nerves. The proportion of macrophages rapidly increased up to 22% at 1 day post-injury and felled to 13.2% at 4 days post-injury. At 14 days post-nerve injury, the proportion of macrophages rapidly decreased to only 2.9%, a level that was similar as that of uninjured nerve (Fig. 3B).

### Changes of axons after sciatic nerve injury

**Fig. 4:**
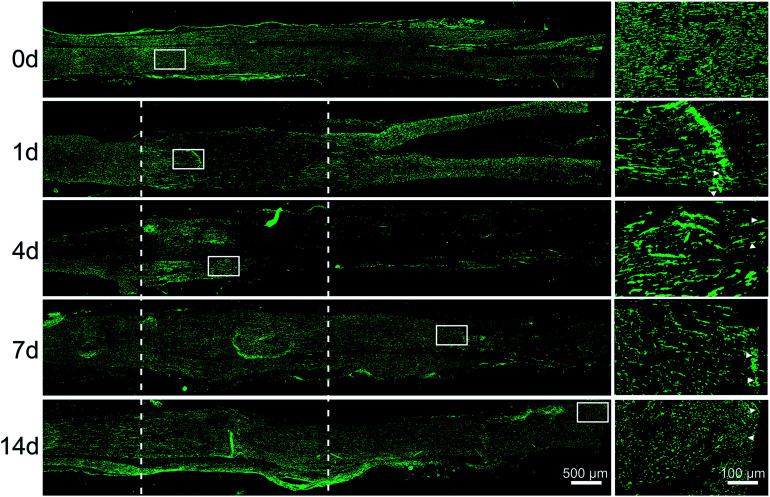
The dynamic distributions of axons following sciatic nerve crush. Immunostaining with anti-NF200 (green) of longitudinal sections of sciatic nerve harvested at 0, 1, 4, 7, and 14 days post-nerve crush, respectively. White dotted line in the left image marked the crushed area (3 mm long). Scale bar: 500 μm. High magnifications of white boxed areas in the left image showed the frontier of axon outgrowth. Scale bar: 100 μm.

Immunostaining with axons marker anti-NF200 showed that axons began to degenerate at crushed nerve segment at 1 day post-injury. Then the distal portion progressively degenerated after 4 days post-sciatic nerve crush, but at the same time, axons of the proximal portion grew forward, the length of regeneration approached the half of crushed nerve segment. After 7 days post-sciatic nerve crush, axons were able to traverse the injury site. At 14 days post-nerve injury, immunocytochemistry with anti-NF200 was conducted to observe that axons could reach the entire distal stump (Fig. 4).

### Let-7 antagomir could enter SCs, fibroblasts, and macrophages

**Fig. 5:**
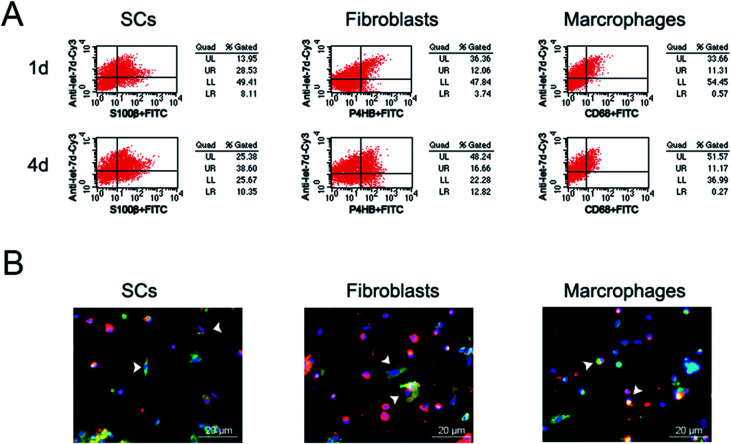
Let-7 entered into SCs, fibroblasts, and macrophages. (A) Flow cytometry showing the proportion of let-7 in SCs, fibroblasts and macrophages at 1 and 4 days post-nerve crush, respectively. (B) Immunostaining with anti-S100β (green), anti-P4HB (green), or anti-CD68 (green) merged with Hoechst 33342 (blue) at 4 days post-nerve crush, respectively. Let-7d antagomir (red) was injected at the crush site. Scale bar: 20 μm.

Previous study^3^ showed that let-7 regulated peripheral nerve regeneration and anti-let-7d could enhance axonal regeneration. However, it remained undetermined that whether let-7 could enter into SCs, fibroblasts, and macrophages, the three main cell types in sciatic nerve stump. Additionally, the influence of let-7 antagomir on SCs, fibroblasts, and macrophages was also unknown. To address this concern, we first observed the influence of let-7d antagomir on the proportion of these three types of cells during sciatic nerve regeneration. Flow cytometry results showed that let-7d antagomir could enter into these three types of cells and influence their proportion (Fig. 5A). The proportion of SCs reached ∼37% at 1 day post-sciatic nerve crush, while up to ∼49% at 4 days post-sciatic nerve crush. The proportion of fibroblasts was ∼16% at 1 day post-sciatic nerve crush, but the amount of fibroblasts rose to nearly two folds. The proportion of macrophages was ∼12% at 1 day post-sciatic nerve crush and there was little change of macrophages at 4 days post-sciatic nerve crush. Compared to control, let-7d antagomir increased the proportion of SCs and fibroblasts, and decreased the number of macrophages at 1 and 4 days after sciatic nerve injury (Fig. 1B, 2B, 3B and 5A). Immunofluorescence results demonstrated that that let-7d antagomir entered into SCs, fibroblasts, and macrophages from the morphological aspect (Fig. 5B).

### The influence of let-7 antagomir on the distributions of SCs, fibroblasts and macrophages

**Fig. 6:**
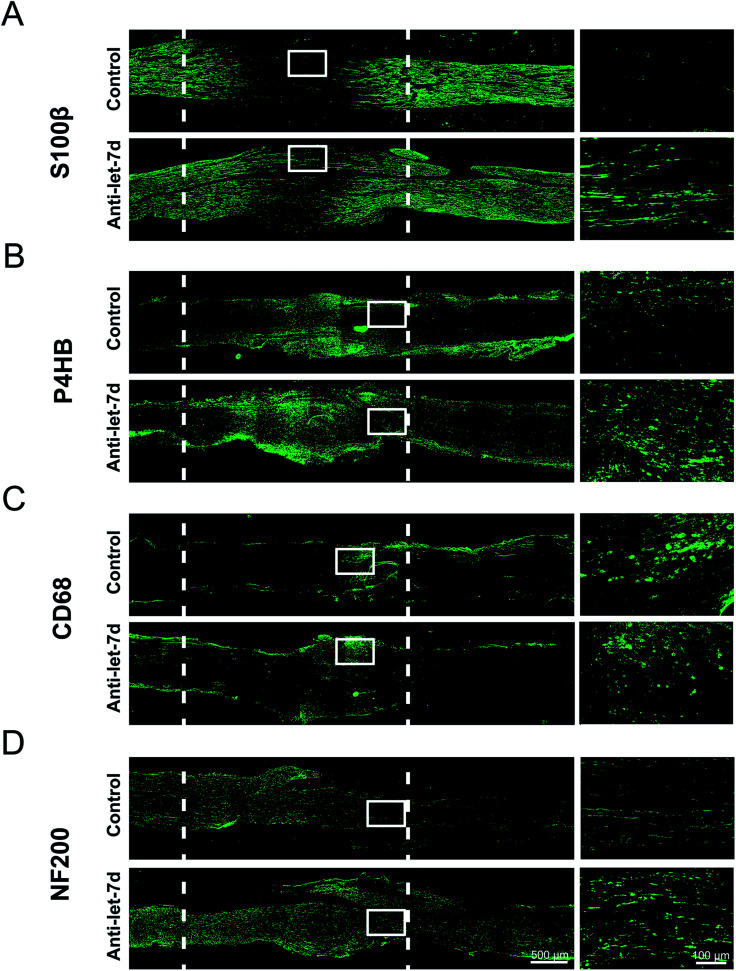
The influence of let-7 antagomir on the distributions of SCs, fibroblasts, macrophages, and axons following sciatic nerve crush. (A) Immunostaining with anti-S100β (green) of longitudinal sections of sciatic nerve harvested at 4 days post-nerve crush. (B) Immunostaining with anti-P4HB (green) of longitudinal sections of sciatic nerve harvested at 4 days post-nerve crush. (C) Immunostaining with anti-CD68 (green) of longitudinal sections of sciatic nerve harvested at 4 days post-nerve crush. (D) Immunostaining with anti-NF200 (green) of longitudinal sections of sciatic nerve harvested at 4 days post-nerve crush. Nerves were divided into two groups to receive the injection of let-7d antagomir (anti-let-7d) or control (control), respectively. White dotted line in the left image marked the crushed area (3 mm long). Scale bar: 500 μm. Right images shown are the higher magnifications in the left image of white boxed areas. Scale bar: 100 μm.

Since let-7d antagomir could enter into SCs, fibroblasts, and macrophages in sciatic nerve, adult rats with sciatic nerve crush were used as an animal model to determine the *in vivo* effects of let-7d antagomir on the distributions of three main cell types during sciatic nerve regeneration. Following nerve injury, the crush site was injected with let-7d antagomir or control, respectively. Immunostaining with anti-S100β or anti-P4HB showed that let-7d antagomir significantly increased the proliferation and migration SCs and fibroblasts at 4 days after sciatic nerve injury, respectively (Fig. 6A and B). And SCs and fibroblasts were distributed in the whole crushed nerve segment. We also found that let-7d antagomir did not affect the distributions of macrophages (Fig. 6C). An elevated axonal growth at 4 days after sciatic nerve injury was also observed after injection with let-7d antagomir (Fig. 6D). These results suggested let-7d antagomir altered the distributions of SCs, increased the numbers of fibroblasts, and thus promoted nerve regeneration.

## Discussion

Peripheral nerve injury leads to a vigorous response from non-neuronal cells in the injured nerve.^8^ The repairmen of an injured peripheral nerve is a complicated and subtle physiological process and requires a comprehensive microenvironment.^41^ SCs, fibroblasts, and macrophages play a significant role in peripheral nerve injury and repair. Therefore, the distributions and changes of SCs, fibroblasts and macrophages are the cellular basis for the treatment of peripheral nerve injury. Compared to transected nerves, crushed nerves exhibit better functional recovery since axons that need to travel only a short distance to reach their target tissue. Considering that rats and humans have the similarity in the distribution of peripheral nerve trunks,^42^ our current study used rat sciatic nerve crush model to investigate the dynamic distributions and changes of SCs, fibroblasts, and macrophages at the different stages during peripheral nerve injury and regeneration.

Under normal circumstances, an uninjured nerve is mainly composed of axons, myelinating SCs, fibroblasts, little residents, inactivate macrophages, and cytokines secreted by these cells.^8,9^ Soon after nerve injury, SCs undergo differentiation, lose their myelinating phenotype, and become repairing cells. Additionally, SCs produce cytokines/trophic factors, proliferate within the basal lamina tubes and align to form bands of Bungner, which provide a supportive substrate and growth factors for axon regeneration.^8,18,20^ Within 48 hours after nerve injury, regeneration-associated genes were up-regulated and SCs begin to proliferate, reaching peak proliferation at around 4 days post-injury.^12^ Inflammatory response plays an essential role in peripheral nerve regeneration by orchestrating interplay between SCs, fibroblasts, macrophages and effector molecules produced by these cells.^13,14^ In the distal nerve stump, SCs recruit immune cells into the injured nerve and then promote axon growth.^12^ Fibroblasts produce extracellular matrix molecules to maintain a structural framework of tissues, play an active role in inflammatory responses and SCs sorting after nerve injury, and release pro-regenerative factors which regulate SCs behavior and neurite outgrowth.^5,18,43^ In additional, fibroblasts can guide the directional migration of SCs and axons from nerve stumps by releasing soluble pro-migratory factors.^9^ Macrophages can remove the inhibitory regeneration signals from myelin debris and pave the way for axonal regrowth to promote peripheral nerve regeneration. They also produce a wide range of factors, such as proteases and growth-promoting factors/cytokines, and stimulate extracellular matrix remodeling to facilitate peripheral nerve regeneration.^19^ Soon after sciatic nerve injury, resident macrophages are increased and activated and the changes of macrophages become more obvious at 3 and 4 days after nerve injury.^19^ Upon contact with myelin surrounding remyelinated axons, activated macrophages are repelled *via* Ras homolog gene family, member A (RhoA) and exit basal laminal tubes.^8^ In our study, we found that at 1 day after nerve crush injury, SCs recruited macrophages which began to accumulate at injured sites and caused acute inflammatory response. Fibroblasts migrated to the crushed nerve segment from the epineurium and perineurium, which might help for preparing SC migration. A lot of macrophages could be observed at 4 days after nerve injury. These macrophages might help to clear myelin debris and axon debris, contributing to the production of an environment that supported SC migration and axon regeneration. At this time point, injured axons began to regenerate along bands of Bungner formed by SCs. The number of fibroblasts was kept increased from 1 to 7 days after injury. Increased amount of fibroblast benefits the migration of SCs and axons. In additional, we found that SCs migrated into middle part from both proximal and distal stumps of injured nerve. When axon traversed the crushed injury site and connected with peripheral targets, macrophages were decreased and little resident macrophages return to inactivated status at 7 days after nerve crush injury, fibroblasts were again gathered in the epineurium and perineurium at 14 days after nerve crush injury. In general, these three cell types, SCs, fibroblasts, and macrophages were correlated and influenced each other, supported axon regeneration, were as the core elements of regulating the regenerative microenvironment. It is worth noting that, in the current study, we used a relative milder injury model (3 mm distance crush) and found significant changes of the proportions of these cells in the sciatic nerve stumps. It is highly likely that the observational difference would be more dramatic after a longer distance crush injury or a more severe transection injury.

In recent years, the applications of miRNAs have been widely put forward and reported,^44,45^ let-7 widely regulates important biological events related to the microenvironment of nerve regeneration, including cell differentiation, proliferation, migration, apoptosis, inflammatory response, axonal regeneration, and myelin formation.^3,26,28–30,46,47^ Therefore, let-7 is as an ideal molecule for regulating regeneration environment to promote nerve regeneration. SCs, fibroblasts, and macrophages are main cells that regulate the microenvironment of nerve regeneration in nerve regeneration. How to integrate the extensive regulatory relationship of let-7 into complex microenvironmental factors in the construction and application of molecular tissue engineering nerve and how to let the external sources be organically combined with the endogenous factors are a series of basic scientific problems that need to be solved and discussed. Therefore, this study firstly investigated the effects of let-7 antagomir on the proportions and distributions of three main cell types. We observed that let-7 antagomir could not only enter into SCs, but also enter into fibroblasts and macrophages during sciatic nerve regeneration. The amount of let-7 antagomir increased with prolongation of the time after nerve injury. On the other hand, immunohistochemistry results further confirmed that at 4 days after sciatic nerve injury in adult rats, let-7 antagomir could promote SC proliferation, SC migration, and axon outgrowth. These results were consistent with previous study in our group.^3^ Inhibition of let-7 could promote the proliferation and migration of SCs and the proliferation of neuroblasts and neural precursor cells (NPCs).^3,30,47^ Thus, an increasing amount of let-7d antagomir might increase the proportion of SCs and fibroblasts by influencing the proliferation and migration of SCs and fibroblasts and produce a suitable microenvironment that be beneficial to nerve regeneration. However, an increasing amount of let-7 antagomir did not increase the proportion of macrophages, even slightly decreased the proportion although the efficiency of anti-let-7d into macrophages was very high. This might because of that macrophages are mononuclear cells and might swallow let-7 when meeting let-7.

## Conclusions

In summary, this paper systematically discussed the dynamic distributions and changes of SCs, fibroblasts and macrophages, the three main cells involved in the microenvironment of nerve regeneration, at different time points after rat sciatic nerve crush injury. In addition, let-7 could modify the regenerative microenvironment by regulating the numbers and distributions of SCs, fibroblasts, or macrophages. Accordingly, our results not only reveal the cell environment basis of microenvironment of peripheral nerve regeneration, but also provide a novel tissue engineering nerve by the application of let-7. A better understanding of these changes can be useful in studying the role of the microenvironment in peripheral nerve regeneration and increasing our understanding of peripheral nerve regeneration, and the application of let-7 into molecular tissue engineering nerve will provide a new insight into peripheral nerve regeneration and suggest a potential therapy for repair of peripheral nerve injury.

## Authors contributions

S. L. and S. Y. conceived and designed the experiments. T. Q., P. W., Q. C., Q. L., H. W., S. W., W. G. and Z. L. performed the experiments. S. L., T. Q., P. W. and H. W. analyzed the data. P. W., Q. C. and Q. L. contributed reagents/materials/analysis tools. T. Q., S. Y., and S. L. wrote the manuscript. All authors read and approved the final manuscript.

## Conflicts of interest

The authors declare that they have no conflict of interest.

## Supplementary Material
